# What is the subtype of dementia in patients with fragility hip fracture?

**DOI:** 10.1371/journal.pone.0265636

**Published:** 2022-04-05

**Authors:** Shigeharu Uchiyama, Fumiki Kamoi, Manabu Tanaka, Itsuo Joko, Kazuo Kasuga, Kenta Suzuki, Naoko Tachibana, Tomoki Kaneko, Naoji Amano

**Affiliations:** 1 Department of Orthopaedic Surgery, Okaya City Hospital, Okaya, Japan; 2 Department of Medical Technology, Radiology Services, Okaya City Hospital, Okaya, Japan; 3 Department of Neurology, Okaya City Hospital, Okaya, Japan; 4 Department of Radiology, Shinshu University School of Medicine, Matsumoto, Japan; 5 Department of Psychiatry, Okaya City Hospital, Okaya, Japan; Medical College of Wisconsin, UNITED STATES

## Abstract

**Introduction:**

Cognitive function is an important factor that affects functional recovery after hip fracture (HipFx) surgery. The literature on the pathophysiology of dementia in HipFx patients is scarce. We performed a differential diagnosis of dementia in HipFx patients using clinical and brain MRI findings.

**Methods:**

This is a prospective study in which brain MRI was evaluated for patients with HipFx for research purposes. One-hundred-and-five HipFx patients (85 females and 20 males) who underwent surgery and were subsequently able to undergo brain MRI at our hospital were evaluated. The mean age was 84 years. The presence of dementia was determined based on clinical findings and whether the patient meets its diagnostic criteria according to the International Classification of Diseases 10th Edition (ICD-10). The differential diagnosis of dementia was made based on brain MRI findings and the dementia diagnostic flow chart published in the Clinical Practice Guideline for Dementia 2017 (Japanese Society of Neurology). The Voxel-based Specific Regional Analysis System for Alzheimer’s Disease (VSRAD) advance 2 diagnostic software was used to evaluate atrophy of the para-hippocampal gyrus.

**Results:**

Fifty-six (53%) patients were clinically diagnosed with dementia according to the ICD-10 criteria. The MRI findings were diverse: Alzheimer’s disease (AD)-type, asymptomatic multiple ischemic cerebral lesions, past symptomatic cerebral infarction or cerebral hemorrhage, Binswanger’s disease (BW)-type, chronic subdural hematoma, disproportionately enlarged subarachnoidal hydrocephalus (DESH), and their combinations thereof. A combination of MRI and clinical findings of dementia patients demonstrated the following distribution of dementia subtypes: AD (n = 20), vascular dementia (n = 33), AD and BW vascular dementia (n = 3).

**Conclusion:**

This study revealed that the brain MRI findings of HipFx patients were diverse. Although vascular dementia is found to be common in this particular population, this could be an incidental finding. Further study is warranted to clarify the specificity of our findings by increasing the number of patients, setting the control, and investigating whether dementia subtypes affect postoperative gait acquisition and fall risk.

## Introduction

Japan’s total population (estimated as of September 15, 2021) has decreased by 510,000 from the previous year, while the elderly population aged 65 and over has increased to its highest ever recorded number at 36.4 million [1]. The percentage of the elderly to the total population is the highest in the world at 29.1%, an increase of 0.3 points from the previous year. By age group, the population aged 80 and over was 12 million (9.6%), an increase of 460,000 (up 0.4 points). With the increase in the geriatric population, the number of osteoporotic hip fractures (HipFx) has also increased with an estimated incidence of approximately 200,000 cases in 2020. Increases in the number of patients from 2009 to 2014 were prominent in the 90 to 94-year-old age group among women and the 85 to 89-year-old age group among men [2].

HipFx is indicated for surgical treatment to facilitate early ambulation. Previous studies have reported that postoperative walking ability of HipFx may not always produce good results, and only about 40 to 60% can regain the same level of walking ability as before injury [3, 4]. Influencing factors for postoperative walking recovery include age, fracture type, pre-injury walking ability, presence of medical complications, cognitive function, and frailty [5, 6]. The reduced walking ability in those with poor recovery is a major cause of bedridden patients, and the cost of long-term care is increasing [7].

On the other hand, the number of patients with dementia is also increasing with the aging of society. It is estimated that approximately 20% of the elderly population (≥ 65 year-old), or 7.3 million people, will develop dementia in 2025 in Japan [8]. Thus, HipFx and dementia are increasing with aging, and the relationship between the two has been a subject of study for more than a decade [9].

Dementia is a risk factor for re-fractures as well as initial HipFx, and the condition often interferes with postoperative rehabilitation and nursing [10, 11]. Therefore, functional recovery is inferior, and the postoperative mortality rate is regarded as being higher than that of patients without dementia [12, 13]. Although HipFx with dementia is expected to continue to increase, many aspects of dementia in HipFx patients remain unclear [14]. This lack of empirical knowledge can significantly affect results, since the evaluation of dementia in HipFx patients is generally performed by screening tests that are not disease-specific, such as the Mini-Mental State Examination (MMSE) and Revised Hasegawa’s Dementia Scale (HDS-R) [5, 15–17]. Although Alzheimer’s disease (AD) is a representative subtype of dementia, there are various other pathological conditions that require different treatment methods and prognosis. Elucidating the type of dementia could enable more effective measures for postoperative rehabilitation and nursing than the status quo, and it may also be possible to change the functional prognosis if deemed treatable. Here, we attempted to diagnose the subtype of dementia in HipFx patients using clinical and brain MRI imaging findings.

## Materials and methods

This was a prospective study, in which brain MRI was evaluated for patients with fragility HipFx for research purposes. The study protocol of this study was approved by our Institutional Ethics Committee (ID number: H29-06).

From November 2017 to April 2019, the number of fragilities HipFx patients who underwent surgery at our hospital was 174. Informed consent was not obtained from 10 patients because 5 patients refused to participate in the study (reasons unknown), 4 patients had a pacemaker, and 1 patient had claustrophobia. In the remaining 164 patients, written informed consent was obtained from the patients and/or their guardians. In 50 of 164 enrolled patients who suffered from severe dementia, written informed consent was obtained by the guardians alone. Fifty-nine of 164 patients were unable to undergo MRI examination due to the following reasons: inability to rest during examination (n = 37), complications of medical illness (n = 12), and transfer to other institutions (n = 10). Thus, a total of 105 patients were able to undergo brain MRI. The age of the 105 eligible patients ranged from 56 to 100 years old with a mean age of 84 years, of which 85 were females and 20 were males. The walking ability before injury was as follows: walk without aids (n = 56), walk with cane or walker (n = 42), and wheelchair (n = 7). The surgical method included fixation with Hansson Pins in 16 patients, Gamma nails in 48 patients, and hemiarthroplasty in 41 patients. The demographic and clinical characteristics of the 105 patients are shown in Table 1.

**Table 1:** Demographic and clinical characteristics of the study population.

Characteristics	
N	105
Age (years) mean ± SD	83.6 ± 9.6
Sex	Female: 85, Male: 20
Height (cm)	150.2 ± 7.8
Weight (kg)	44.5 ± 9.9
BMI(kg/m^2^)	19.6 ± 3.6
BMD (%YAM), contralateral hip	58 ± 18 (n = 83)
Preinjury walking ability	Walk without aids: 56 Cane or walker: 42 Wheelchair: 7
Fracture classification	Femoral neck fracture: 57 Trochanteric fracture: 48
Side	Right: 59, Left: 46
Pre-existing vertebral fracture	None: 35, One: 12, Two: 16, Three or more: 42
Medical History	None: 7, Hypertension: 44, Hyperlipidemia: 13, Contralateral hip fracture: 4, Cardial: 16, Pulmonary: 4, Cerebrovascular: 22, Diabetes: 10, Osteoporosis: 17, Parkinson’s disease: 6, Dementia: 14, Cancer: 9, Epilepsy: 4.
Preinjury osteoporotic treatment	Yes: 15 (Bisphosphonate: 6, SERM: 3, Denosumab: 1, Eldecalcitol: 8)
Nursing home before admission	Nursing home: 22, Own home: 83 (Living alone: 8)
Surgery type	Hansson pin: 16 Gamma nail: 48 Hemiarthroplasty: 41

*BMI* Body mass index, *BMD* Bone mineral density, *YAM* Young adult mean,

BMD as normal (BMD ≥ 80% of YAM), osteopenia (70% ≤ BMD < 80% of YAM) or osteoporosis (BMD < 70% of YAM).

*SERM* Selective estrogen receptor modulator.

### Clinical diagnosis of the presence of dementia

The presence of dementia was evaluated by a team of orthopedists, a neurologist, and a psychiatrist. A comprehensive assessment was conducted to determine whether the criteria of ICD-10 Diagnostic Criteria for Dementia (any cause) [18] (S1 Appendix) were met. The assessment was based on responses to interviews conducted during postoperative ward rounds by orthopedists (4 times a week up to 1 month after surgery), physical findings, nursing records, interviews with family members about dementia at first visit, and the HDS-R examined by a nurse.

At our hospital, the psychiatrist (NA), who is a dementia specialist, regularly provides guidance for ward nurses on how to use HDS-R. An HDS-R score of 20 or lower was defined as suspected dementia. HDS-R was performed twice, the first time within one week of admission and the second time at 1 month after surgery to evaluate reliability and effects of postoperative delirium.

In patients with delirium, the second test scored higher than the first one. Thus, the second test value was used for dementia evaluation. The symptoms of delirium could be determined by the clinical course of one month after surgey. Evaluation of patients were first made by orthopedists and ward nurses. In patients with suspected postoperative delirium, the diagnosis and treatment were conducted by a psychiatrist (4 patients). The final diagnosis for the presence of dementia was determined by the neurologist (NT) after she reviewed the medical records of all patients.

### Brain MRI findings including early Alzheimer’s Disease (AD) diagnostic software Voxel-based Specific Regional Analysis System for Alzheimer’s Disease (VSRAD) advance 2

A brain MRI was taken when the condition of the patients was stable within 1 month after surgery.

A GE Signa 1.5T HDxt Version 23 (GE Healthcare, Milwaukee, Wisconsin) with a head/neck/spine (HNS) coil was used as an imaging system. For VSRAD, the following conditions were used: image sequence, 3D Fast SPGR (sagittal section); TR, 11.2 ms; TE, 5.1 ms; FOV, 250 mm; FA, 25 degrees; TI, 400 ms; matrix, 256×256 pixels; slice thickness, 1.5 mm; BW, ±15.63kHz/FOV; NEX, 1; number of slices, 1 slab (120~130 slices).

VSRAD is an image processing and statistical analysis software for reading the degree of medial temporal atrophy from MRI images that is characteristic of early AD [19]. It has been confirmed that in early AD including the prodromal stage, the discrimination rate is 90% or more compared to the healthy elderly [19]. AD was suspected to have a Z score of 2.0 or higher in the volume of interest (VOI; obtained from the hippocampus, tonsils, and olfactory area). The Z score is a statistical comparison between a subject image and a healthy person image. As a result, it is a value indicating how much the standard deviation is separated from the mean value. A Z score of 0 to 1 indicates almost no para-hippocampal atrophy, 1 to 2 indicates slight atrophy of the para-hippocampal gyrus, 2 to 3 indicates considerable para-hippocampal atrophy, and 3 or more indicates strong para-hippocampal atrophy.

The criteria for interpretation of the findings of the lesions in the brain are shown below. Two examiners (neurologist [NT] and neuroradiologist [TK]) independently recorded the lesions of 105 cases of brain MRI. The number of lesions was not always limited to one lesion in each patient. Interrater reproducibility was calculated for each lesion using the kappa value.

- AD type: significant atrophy of the para-hippocampal gyrus, which is considered to be positive when the Z-score is 2.0 or more in the VSRAD analysis. However, when low signal intensity lesions more than 1cm diameter exists in the brain, the calculation of Z score is not reliable. Thus, atrophy of that region was evaluated qualitatively in those particular cases.
- Multiple ischemic cerebral lesions: multiple high signal intensity lesions in the white matter that spread moderately thicker around the ventricles (Fazekas 2) [20].
- Binswanger’s disease(BW) type: extensive white matter lesions (Fazekas 3).
- Past cerebral infarction or hemmorhage: FLAIR low intensity lesion surrounded by high intensity (cerebral infarcion) or low signal intensity lesion in T2*-weighted image (hemorrhage).
- DESH (disproportionately enlarged subaracahnoid space hydrocephalus): enlargement of the ventricular and the Sylvian fissure. The subarachnoid space of the parietal region (higher fornix and longitudinal fissure of the cerebrum) is narrowed, but the basal cistern and Sylvian fissure are expanding.
- Chronic subdural hematoma: crescent-shaped lesions between the dura and arachnoid
- Normal aging: no medial temporal atrophy (Z-score less than 2.0), no or subtle white matter lesions (Fazekas 0 or 1), or no other lesions.

### Differential diagnosis of dementia

Patients who were clinically diagnosed with dementia were processed to differential diagnosis of dementia. The diagnosis was made based on MRI findings and the dementia diagnosis flow chart published in the Clinical Practice Guideline for Dementia 2017 (Japanese Society of Neurology) [21] (S2 Appendix).

In particular, the differentiation between AD and vascular dementia was performed as follows:

AD was diagnosed when the Z score in VSRAD was 2 or more, without significant ischemic lesions in MRI. Clinically, if the patient had the following characteristics, the diagnosis of AD is more preferable to vascular dementia: 1) insidious onset and slow progression; 2) onset due to recent memory impairment; 3) further progression with the addition of disorientation, executive dysfunction, and visuospatial disorders; 4) presence of psychiatric symptoms such as apathy and/or depression, decreased insight, in addition to characteristic interpersonal behavior such as saving appearance responses; 5) noticeable cognitive dysfunction other than memory in cases of presenile onset, such as aphasia and impaired visuospatial cognition; 6) no significant local neurological symptoms from the early stages of the disease [22].

Vascular dementia was diagnosed when the Z score in VSRAD was less than 2 points with significant ischemic changes in brain MRI. In addition to cognitive dysfunction, if the patient had the following associated symptoms and complications, vascular dementia was more likely than AD: 1) gait disorder, fall, dysuria, pseudobulbar palsy, and/or depression; 2) in addition to stroke, ischemic heart disease and peripheral arterial disease may be complicated as systemic angiopathy [23].

Since vascular dementia is known to merge with AD, it is sometimes difficult to make a clear diagnosis of dementia. Therefore, we carefully referred to the daily medical records to focus attention on a differential diagnosis of dementia. The neurologist made the final clinical diagnosis after all patient data were available in June 2019.

## Results

Forty-four patients scored 21 or higher in HDS-R, and 61 patients scored lower than 21; however, 56 (53%) were clinically diagnosed with dementia that met ICD-10.

The number of patients with VSRAD exhibiting a Z-score (VOI atrophy degree) of 2.0 or higher (AD on imaging) was 43 (41%), and those 2.0 or lower was 62 (59%).

**Fig 1:**
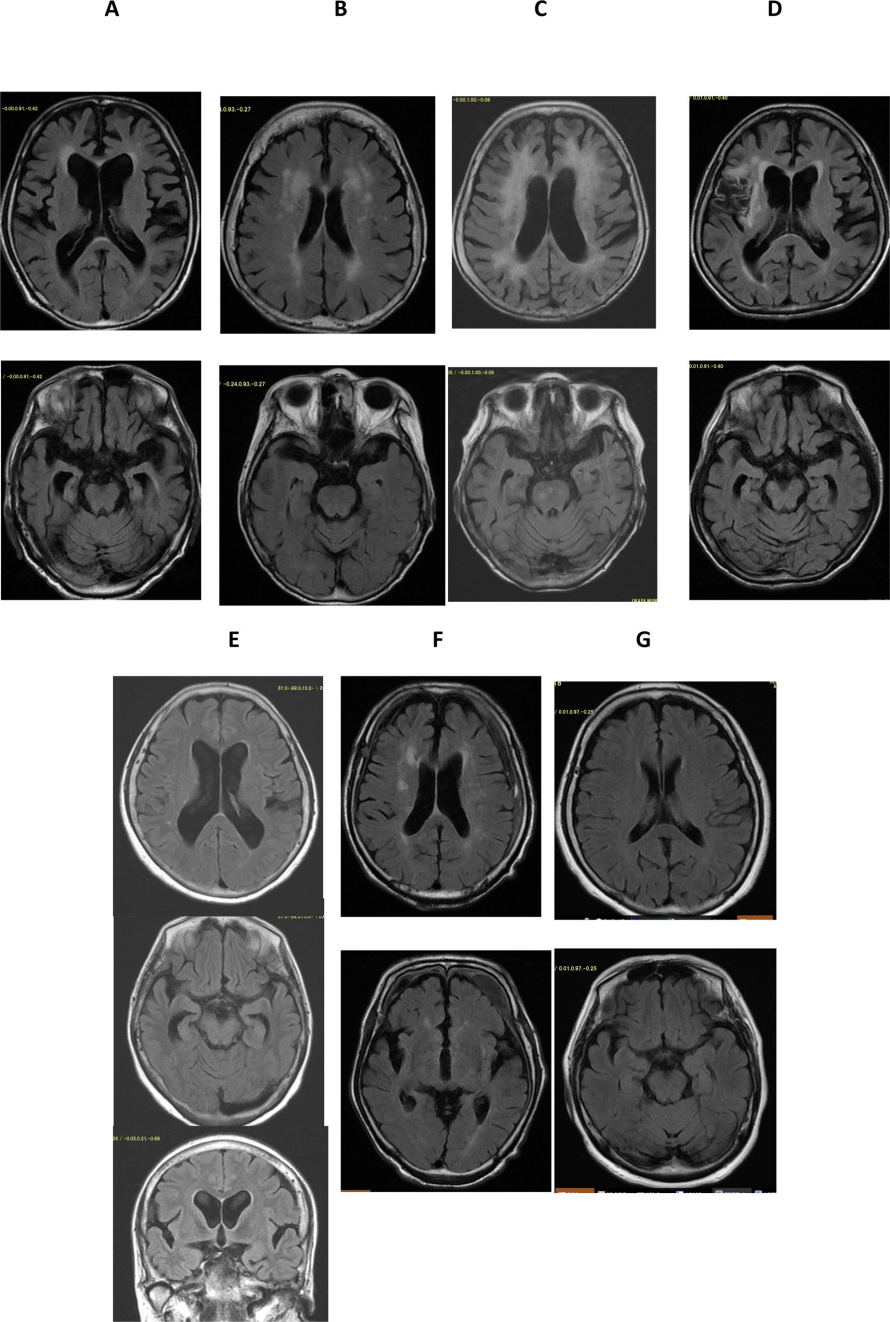
Representative brain MRI lesions in patients with HipFx. T2-FLAIR axial image. A. Alzheimer’s disease-type: significant atrophy of the para-hippocampal gyrus. B. Asymptomatic multiple ischemic cerebral lesions: multiple high signal intensity lesions in the white matter. C. Binswanger’s disease-type: extensive white matter lesions. D. Past symptomatic cerebral infarction: large low signal intensity lesion in the right temporal lobe. E. DESH (disproportionately enlarged subaracahnoid space hydrocephalus): enlargement of the ventricular and the Sylvian fissure F. Chronic subdural hematoma: crescent-shaped lesions between the dura and arachnoid G. Normal aging: no atrophy or no white matter lesions.

MRI findings were diverse, and the representative pathological conditions observed by the neurologist are shown in Fig 1. The MRI findings of 105 patients are shown in Table 2.

**Table 2:** MRI findings of 105 patients.

MRI findings	Dementia patients n = 56	Non-dementia patients n = 49	Total n = 105
Asymptomatic multiple ischemic cerebral lesions	9	18	27
BW type	11	1	12
AD type	10	4	14
AD type + asymptomatic multiple ischemic cerebral lesions	9	7	16
Past symptomatic cerebral infarction / cerebral hemorrhage	5	2	7
AD type + BW type	5	0	5
BW type + past symptomatic cerebral infarction / cerebral hemorrhage	2	0	2
BW type + chronic subdural hematoma	1	0	1
AD type + chronic subdural hematoma	1	0	1
BW type + DESH	1	0	1
Chronic subdural hematoma	1	0	1
AD type + past symptomatic cerebral infarction / cerebral hemorrhage	1	0	1
DESH	0	1	1
Normal aging	0	16	16

*AD* Alzheimer’s disease, *BW* Binswanger’s disease, *DESH* disproportionately enlarged subarachnoid space hydrocephalus.

Normal aging was seen only in 16 patients (15%). Vascular pathology (cerebral infarction/hemorrhage, multiple ischemic cerebral lesions, BW type) was observed in 72 patients (69%), of which 44 (79%) were dementia patients, and 28 (57%) were non-dementia patients.

The kappa value of each lesion for interrater reproducibility evaluation was as follows:

AD type, 1.000; multiple ischemic cerebral lesions, 0.60; BW type, 0.753; past cerebral infacrtion or hemmorhage, 0.593; DESH, 0.386; chronic subdural hematoma, 0.581; normal aging, 0.964. Overall, the concordance rate of the interpretative findings of 105 patients for the two examiners was 73%.

**Fig 2:**
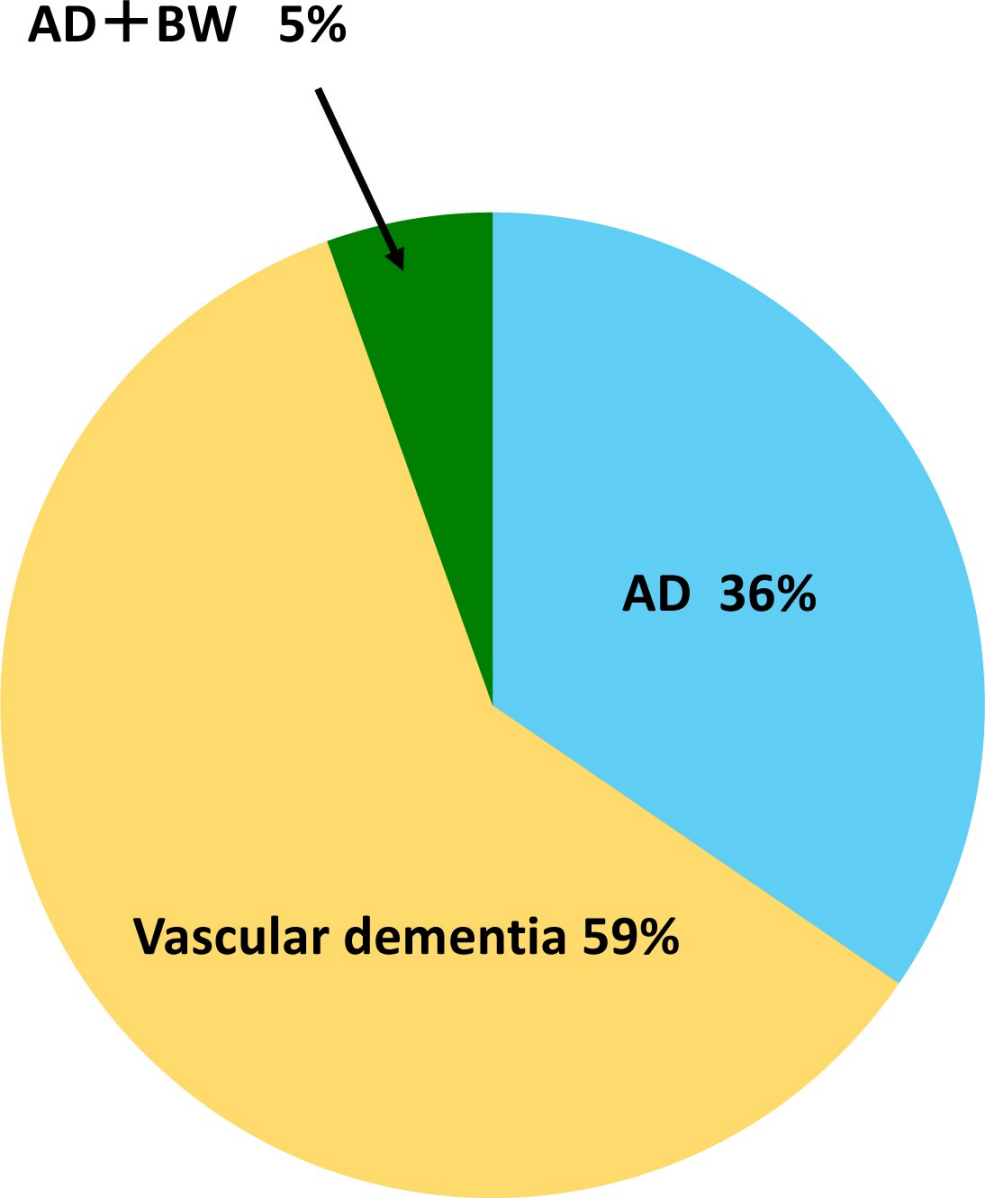
Differential diagnosis of 56 hip fracture patients diagnosed as having dementia. AD: 20 (36%), vascular dementia: 33 (59%), AD + BW: 3 (5%). Vascular dementia as well as AD is commonly seen in HipFx patients. AD: Alzheimer’s disease, BW: Binswanger’s disease.

When MRI and clinical findings were combined, the distribution of dementia subtypes were as follows: AD (n = 20), vascular dementia (n = 33), and AD + BW vascular dementia (n = 3) (Fig 2).

## Discussion

HipFx is the most serious osteoporotic fracture in the elderly. Not only is surgery a high risk, but there are also many associated social problems such as restrictions on postoperative functional recovery and soaring medical costs. Osteoporosis is not the only cause of hip fractures, and previous reports have also emphasized the presence of dementia as a factor that contributes to a higher risk of sustaining hip fractures [24, 25]. According to recent reports, the incidence of dementia in HipFx patients ranges from 10 to 66% [5, 15–17, 26]; however, it is believed that there are differences depending on the method of detecting dementia. In reports from Japan, dementia is evaluated by screening tests such as MMSE, AD8 (Eight-item Interview to Differentiate Aging and Dementia), or HDS-R [15–17]. Since all of these are retrospective studies, details such as the prevalence of dementia subtypes are unknown. This study found that the brain MRI findings of HipFx patients were diverse, and vascular dementia could be a common cause of dementia as well as AD.

In recent years, MRI imaging has been used as an auxiliary diagnostic tool for early dementia [19]. The classification of dementia is divided into AD, vascular dementia, and Lewy body dementia in addition to other subtypes. AD is the most frequent of these and account for approximately 68% in the general population [27]. Since it is known that atrophy of the medial temporal region appears characteristically in early AD, VSRAD has made it possible to analyze and convert the morphological information into auxiliary diagnostic information [19]. Although the need for diagnostic imaging has been suggested for the evaluation of dementia in HipFx [16], there have been no reports on differential diagnosis. In our study, the VSRAD analysis of the Z-score helped us to make a differential diagnosis of AD and vascular dementia. A Z-score of 2.0 or more was more likely to be diagnosed with AD, but a Z-score of less than 2.0 was unlikely to be diagnosed with AD. Cases below the 2.0 threshold could be diagnosed with vascular dementia if significant ischemic changes were observed in brain MRI.

In terms of the diagnostic reliability of dementia, the validity and reproducibility of the flow chart have not been verified. However, the HDS-R used in the process had a Cronbach’s alpha coefficient of 0.90, a correlation of 0.94 with MMSE, and a sensitivity of 90% for suspected dementia. The specificity was reported to be 82% [28]. Regarding the interpretation of brain MRI, the concordance rate between the neuroradiologist and the neurologist was 73%, and the kappa value regarding the evaluation of individual findings was moderate to perfect agreement, except for DESH. Based on the above, it could be determined that the reliability of this dementia diagnosis (including subtype diagnosis) is acceptable.

Vascular dementia is accompanied by asymptomatic multiple cerebral ischemic lesions and cerebral leukoaraiosis such as BW type, and these lesions are considered to reduce ADL such as depression, gait disturbance, and visual impairment [29]. In addition, most HipFx result from falls, and easy falls due to muscle weakness and gait disturbance is a problem. The brain MRI of HipFx patients revealed in this study showed vascular pathology (cerebral infarction/hemorrhage, multiple ischemic cerebral lesions, BW type) not only in dementia patients (79%) but also in non-dementia patients (57%), and these brain lesions may increase the risk of fracture.

This information may be useful when considering postoperative rehabilitation and prevention of re-fracture in HipFx patients. Advances in diagnostic imaging such as MRI may include treatable conditions such as normal pressure hydrocephalus, chronic subdural hematoma, and brain tumors. If the condition is vascular, it is considered possible to recover and maintain rehabilitation functions such as walking training while preventing the recurrence of vascular disorders, falls, and pneumonia by medical treatment.

Due to the lack of control and limited number of the patients, it is not possible to make a rigorous determination as to whether MRI findings are specific to HipFx patients. However, the literatures in Japan that describe the brain MRI findings of people of the same age who lead normal lives may be different from those of our patients. For example, large cerebral hemorrhage and cerebral infarction lesions, BW type white matter lesions, DESH, and chronic subdural hematoma are not observed, even if T2 high signal intensity lesions around the ventricles are frequently observed at 72% [30]. Another Japanese study indicated that periventricular hyperintensity lesions and small lacunar lesions were seen in 27.3% and 36.4% of normal elderly patients aged more than 75 years old, respectively. No other significant lesions were reported in that population [31]. Considering the above, the brain MRI findings in this study could be different from those of elderly people living in good health.

The limitations of this study are as follows: Thirty-seven patients with dementia were excluded because they were unable to undergo MRI imaging due to cognitive disorientation or their inability to remain still during examination. Thus, the dementia patients analyzed in this study do not represent all the dementia patients with HipFx. Only the orthopaedic surgeons actually met all the patients and a psychiatrist saw the patients with suspected delirium. The neurologist did not examine the patient during the interview. Furthermore, due to wide variety of brain MRI findings, it was difficult to determine which was predominant when the pathologies are mixed. Thus, prevalence of AD or vascular dementia could be different from our present data. Finally, due to the lack of control and the small number of patients, MRI findings in our patients could be incidental. Despite these limitations, this study is the first to diagnose dementia subtype by performing brain MRI of patients with HipFx and provides new data in understanding these patients.

In conclusion, this study revealed that the brain MRI findings of HipFx patients were diverse. Although vascular dementia was found to be common in this particular population, this could be an incidental finding. Further study is warranted to clarify the specificity of our findings by increasing the number of patients, setting the control, and investigating whether dementia subtypes affect postoperative gait acquisition and fall risk.

## Supporting information

S1 Appendix (PDF)

S2 Appendix (PDF)
