# Supplementary material for: What is the subtype of dementia in patients with fragility hip fracture?
Source: PLoS One. 2022 Apr 5;17(4):e0265636. doi: 10.1371/journal.pone.0265636 (PMC8982891; doi:10.1371/journal.pone.0265636)
Supplement: S1 Appendix — (PDF) [file pone.0265636.s001.pdf]

Appendix 1

ICD-10 Diagnostic Criteria for Dementia (any cause)

G1.1 Decline in memory, esp. learning new information; both verbal and nonverbal material

G1.2 Decline in other cognitive abilities; deterioration in judgment and thinking

G2 Preserved awareness of environment

G3 Decline in emotional control or motivation, or change in social behaviour, eg emotional lability ,irritability, apathy, coarsening of social behaviour

G4 Criterion G1 clearly present for at least 6 months
