# Supplementary material for: What is the subtype of dementia in patients with fragility hip fracture?
Source: PLoS One. 2022 Apr 5;17(4):e0265636. doi: 10.1371/journal.pone.0265636 (PMC8982891; doi:10.1371/journal.pone.0265636)
Supplement: S2 Appendix — (PDF) [file pone.0265636.s002.pdf]

Flow chart of diagnosis of dementia

Clinical Practice Guideline for Dementia 2017 (Japanese Society of Neurology)

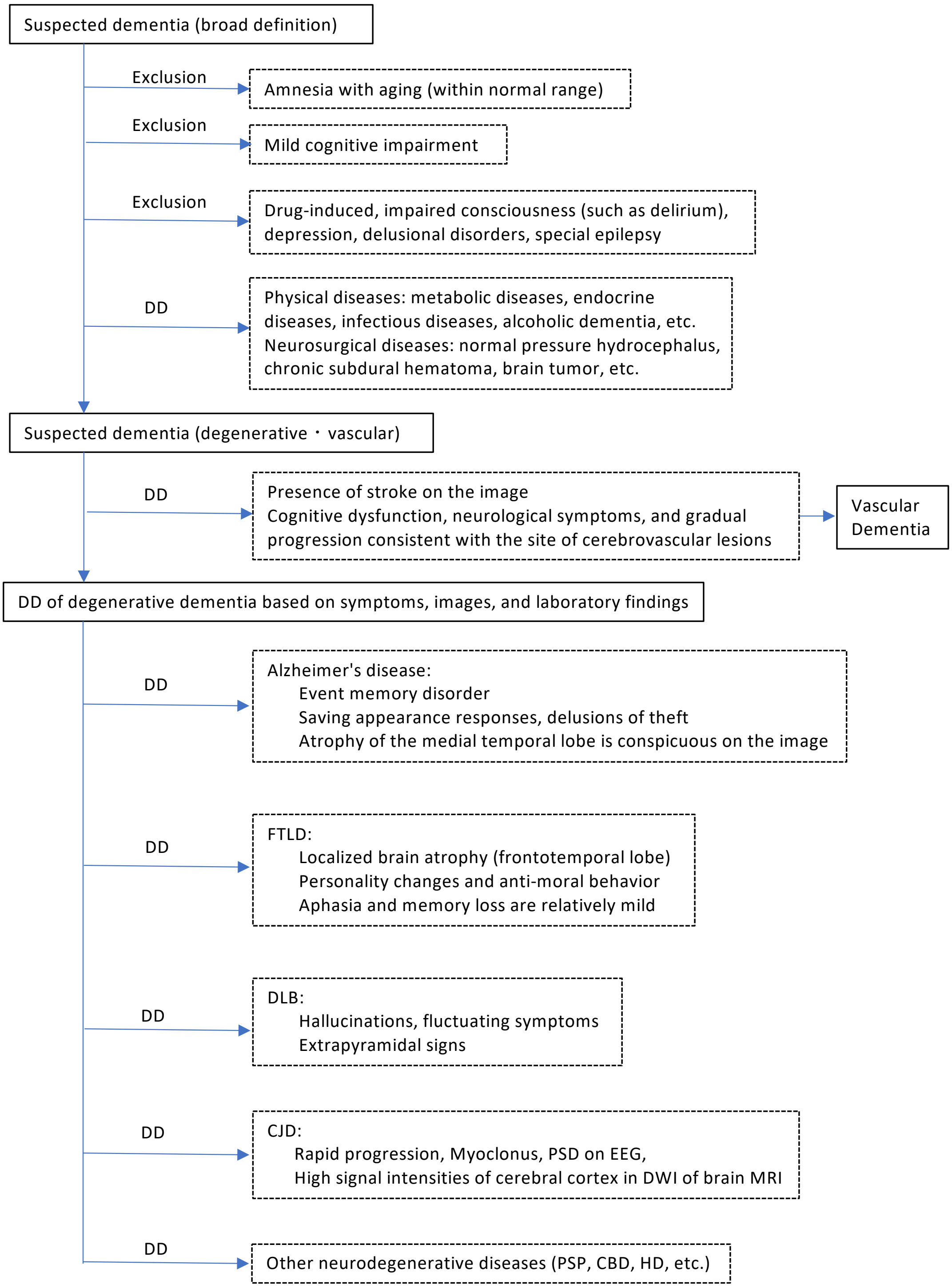

DD: differential diagnosis, DWI: diffusion weighted image,
FTLD: frontotemporal lobar degeneration,
DLB: dementia with Lewy bodies,
CJD: Creutzfeldt-Jakob disease,
PSD: periodic synchronous discharge,
PSP: progressive supranuclear palsy,
CBD: corticobasal degeneration,
HD: Huntington's disease
